# Psychometric evaluation of a decision quality instrument for medication decisions for treatment of depression symptoms

**DOI:** 10.1186/s12911-021-01611-w

**Published:** 2021-08-27

**Authors:** Suzanne Brodney, K. D. Valentine, Karen Sepucha

**Affiliations:** 1 Informed Medical Decisions Program, Massachusetts General Hospital, 100 Cambridge Street, 16th Floor, Boston, MA 02114 USA; 2 Health Decision Sciences Center, Massachusetts General Hospital, 100 Cambridge Street, 16th Floor, Boston, MA 02114 USA

**Keywords:** Shared decision making, Patient centered care, Quality measurement, Depression symptoms

## Abstract

**Background:**

A high quality treatment decision means patients are informed and receive treatment that matches their goals. This research examined the reliability and validity of the Depression Decision Quality Instrument (DQI), a survey to measure the extent to which patients are informed and received preferred treatment for depression.

**Methods:**

Participants were aged 18 and older from 17 US cities who discussed medication or counseling with a physician in the past year, and physicians who treated patients with depression who practiced in the same cities. Participants were mailed a survey that included the Depression-DQI, a tool with 10 knowledge and 7 goal and concern items. Patients were randomly assigned to either receive a patient decision aid (DA) on treatment of depression or no DA. A matching score was created by comparing the patient’s preferred treatment to their self-reported treatment received. Concordant scores were considered matched, discordant were not. We examined the reliability and known group validity of the Depression-DQI.

**Results:**

Most patients 405/504 (80%) responded, 79% (320/405) returned the retest survey, and 60% (114/187) of physicians returned the survey. Patients’ knowledge scores on the 10-item scale ranged from 14.6 to 100% with no evidence of floor or ceiling effects. Retest reliability for knowledge was moderate and for goals and concerns ranged from moderate to good. Mean knowledge scores differentiated between patients and physicians (M = 63 [SD = 15] vs. M = 81 [SD = 11], *p* < 0.001), and between patients who did and didn’t receive a DA (M = 64 [SD = 16] vs. M = 61 [SD = 14], *p* = 0.041). 60.5% of participants received treatment that matched their preference. Based on the multivariate logistic regression, ‘avoiding taking anti-depressants’ was the only goal that was predictive of taking mediation (OR = 0.73 [0.66, 0.80], *p* < 0.01). Shared Decision Making Process scores were similar for those who matched their preference and those who didn’t (M = 2.18 [SD = 0.97] vs. M = 2.06 [SD = 1.07]; t(320) =  − 1.06, *p* = 0.29). Those who matched had lower regret scores (matched M = 1.72 [SD = 0.74] vs. unmatched M = 2.32 [SD = 0.8]; t(301) =  − 6.6, *p* < .001).

**Conclusions:**

The Depression DQI demonstrated modest reliability and validity. More work is needed to establish validity of the method to determine concordance.

*Trial registration*: NCT01152307.

**Supplementary Information:**

The online version contains supplementary material available at 10.1186/s12911-021-01611-w.

## Background

Guidelines from the American Psychological Association [1] and the American College of Physicians [2] for adults with major depressive disorder recommend a shared decision making (SDM) approach to help patients decide between psychotherapy or medication as initial treatment. In SDM, the clinician typically communicates the available treatment options and the benefits and harms of those options, the patient communicates their treatment preferences to inform the clinician about what matters to them and together, the patient and clinician decide on the treatment [3]. Despite guidelines recommending its use, SDM for depression treatment is underutilized [4–7].

The goal of SDM is to improve the quality of treatment decisions [8]. In order for a decision to be considered ‘high quality’ two conditions must be met: (1) patients need to be informed, meaning they understand the basic facts about their condition and their treatment choices, and (2) the treatment that the patient receives should match their goals [9]. Data from past national surveys indicates that people with depression are not informed about depression or its treatments [10–12]. Patients with depression who had less formal education were less informed, less involved in the decision making process and less likely to report they would make the same decision again [13]. When treatment decisions between patients and clinicians are not shared, patients are more likely to receive treatment that does not match their values [14]. Promoting SDM may result in better quality decisions, but there is no validated measure of the quality of depression treatment decisions.

Sepucha and colleagues have proposed a method for developing decision quality instruments that measure knowledge about treatment options and concordance (the match between patients’ goals and treatments) and have developed instruments for several common surgical decisions, including breast cancer surgery, joint replacement for knee and hip, and treatment for herniated disc [15–18]. The conceptual framework driving the development process describes knowledge-based and patient-centered care as the goal for shared decision making. In order to achieve a high-quality decision, patients need to be informed about their condition and treatment options, and they need to receive treatments or tests that match their preferences. The present study aims to extend this work to the context of depression by describing the development and evaluation of the psychometric properties of the Depression-Decision Quality Instrument (Depression-DQI) using data from two samples: (1) a retrospective survey of patients who made a decision about treatment for depression symptoms within the past year, and (2) a multidisciplinary group of clinicians who treat patients with depression symptoms.

## Methods

### Depression decision quality instrument (DQI) development

The development of the Depression-DQI followed an established protocol that has been used to develop decision quality instruments for other conditions [15–19]. The development process began by reviewing clinical evidence regarding treatment options and was supplemented with findings from focus groups with patients who had been diagnosed with depression. Based on the qualitative work, it was discovered that while patients often considered both medications and counseling/therapy for treatment of their depression, they did not always do this simultaneously. In other words, patients discussed the decision about whether to start or stop medication separately from the decision about whether to start or stop counseling/therapy. As a result, we designed questions that consider these decisions separately in the survey. A set of candidate facts was developed that measure how informed the patients are about treatment options for depression, and a separate set of goals and concerns were developed that measure what matters to patients to help determine if patient treatment preferences match their goals and concerns. In 2008 the facts and goals were reviewed and rated by a convenience sample of patients with depression symptoms (n = 40) and a multidisciplinary group of clinical experts (n = 23). A draft of the Depression-DQI was evaluated with cognitive interviews (n = 5) with patients with depression. Patients discussed their understanding of each question and reasoning behind their responses in order to ensure that the items were being interpreted and answered appropriately. Questions were edited to improve understanding, such as replacing the word medication with medicine and adding the term counseling and therapy instead of just counseling.

### Samples and procedures

In 2010, two samples were surveyed to provide evidence on the performance of the Depression-DQI.

The retrospective patient sample included adults 18 years and older who had discussed medication or counseling for treatment of depression with a health care provider within the past year. Patients were recruited through online and newspaper ads in 17 U.S. cities (Atlanta, Baltimore, Boston, Chicago, Dallas-Ft. Worth, Denver, Detroit, Ft. Myers, Houston, Los Angeles, Minneapolis, New York, Phoenix, Portland, Raleigh-Durham, San Francisco, Washington DC). All respondents were screened by phone for eligibility then respondents were randomized to receive either a depression patient decision aid (DA) or no DA. The DA, *Coping with Symptoms of Depression,* is a 35-min DVD and 48-page booklet that discusses a wide range of treatment options produced by the Foundation for Informed Medical Decision Making and Health Dialog ©2008. All participants were mailed study materials; non-responders were sent a reminder survey about two weeks after the initial mailing. All responders were contacted by mail to participate in a retest survey four weeks after completing the initial survey. Patients received incentives if they completed the survey ($30 for DA, $20 for no DA, and $10 for retest). The study protocol was approved by the Institutional Review Board at Massachusetts General Hospital and the Center for Survey Research at University of Massachusetts Boston.

The physician sample included primary care physicians and psychiatrists from the American Medical Association (AMA) who practiced in the same 17 cities as the retrospective patient sample. A sample of 100 primary care clinicians and 100 psychiatrists was selected. Each provider was mailed a survey with a $20 incentive. A reminder call was made two weeks after the mailing, and a mailed reminder was sent two weeks after the reminder call. The provider study protocol was approved by the Institutional Review Board at Massachusetts General Hospital.

### Measures

The patient sample completed the Depression-DQI and answered survey questions about demographics, treatment preference, treatment received, top two goals and concerns, decision regret, shared decision making, physical and mental health status, and subjective numeracy.

*Depression DQI*: The Depression DQI included 10 knowledge questions and seven goals and concerns which were rated on an 11-point importance scale (0 [Not at all important to me] − 10 [Extremely important to me]). A total knowledge score was calculated by summing correct responses. Missing knowledge responses were scored as incorrect. A total knowledge score (0–100%) was calculated for all respondents who answered > 50% of the items and was standardized by dividing the number of correct responses by the number of items.

*Matching score*: A matching score was created by comparing the patient’s preferred treatment to the treatment received. Treatment Preference was determined by the question: “Did you want to take antidepressant medicine to treat your depression?” with response options of yes, no or unsure. Treatment Received was determined by the question: “In the last 12 months, have you used anti-depressant medicine to treat your depression?” with response options yes or no. If a patient preferred to take medication and received medication, or if the patient preferred not to take medication and didn’t receive medication, these were categorized as matching. Patients who did not receive their preferred treatment or were unsure were categorized as not matching. A similar set of items was asked regarding desire to receive depression counseling or therapy and current use of depression counseling or therapy; a similar match variable was also created. These counseling/therapy items were assessed alongside the medication items but resulted in similar conclusions and thus are located in the Additional file 3 for clarity of presentation.

*Shared decision making (SDM) process scale*: Patients completed the SDM Process scale, which included four questions that measured the interaction between the patient and the provider [20]. Points were summed to generate a total SDM Process Scale score (range 0–4) with higher scores indicating greater SDM was present.

*Decision regret*: A single item assessed whether patients would choose the same treatment again [18, 21]. The question asked, “If you had to do it again, do you think you would make the same decision about how to treat your depression?” Response options included definitely yes, probably yes, probably no and definitely no. Points were assigned as follows: definitely yes = 1 point, probably yes = 2 points, probably no = 3 points and definitely no = 4 points. Higher scores indicate greater regret.

*Provider survey*: Physicians completed a questionnaire that included the same 10 knowledge questions patients responded to, demographics, specialty type, years in practice and number of patients seen per year with depression. Physicians were asked to categorize each of the knowledge items as essential, important, or not important. They also provided feedback on how well they thought the items represented key facts (extremely well, very well, somewhat well, not at all well).

### Statistical analyses

*Patient and provider characteristics*: The overall patient sample is described and differences between those who received the DA and those who did not were analyzed with two sample *t*-tests for continuous variables and chi-square analyses for categorical variables. The overall provider sample separately describes primary care providers and psychiatrists.

#### Item retention and deletion

*Knowledge*: A priori criteria were established to examine the knowledge questions and determine which items to revise or delete. These criteria were based on level of difficulty (> 80% of patients answered correctly or < 50% of providers answered correctly), problematic formats (> 5% with missing data or multiple responses), item to total correlations (< 0.3), or content validity (< 25% of providers feel the question is essential or > 75% of providers considered an item as essential).

*Goals and concerns*: The criteria for revising or deleting the goals and concerns included evidence of a ceiling or floor effect (skew > 1), more than 5% of missing data, multiple responses or poor participant response, ability to discriminate between those choosing or not choosing medication, and goals that did not get rated by the patient as the first or second most important.

#### Acceptability and feasibility

Acceptability was examined using response rates. Feasibility was examined using rates of missing data, multiple responses or incorrect skips for individual items and total scores.

#### Reliability

Test–retest reliability was assessed with the intra class correlation coefficient (ICC (2k)) with 95% confidence intervals (CIs) for the knowledge score, goals and concerns, treatment preference, and for the SDM Process scale. Responses over the four-week window were not expected to change for the retrospective sample and the target was ICC ≥ 0.70. Internal consistency was not calculated as the knowledge items, goals and concerns and SDM Process scale are not one underlying construct.

#### Validity

Hypothesis testing was used to provide evidence of validity for the knowledge items and the goals and concerns. Several hypotheses were established a priori.

*Known group validity hypotheses*: We hypothesized that mean knowledge scores would be higher for physicians than patients and that mean knowledge scores for patients in the DA group would be higher than the scores for patients who did not get a DA (both tested with two sample *t*-test).

For the goals and concerns, we first used univariate logistic regression models to identify if any goals and concerns were predictive of taking medication. Those goals and concerns that were significant (*p* < 0.15) were used in a multivariate model to generate a predicted probability of taking medicine. Then, we tested the hypothesis that patients who stated a preference for medicine would have a higher predicted probability of taking medicine compared to those who did not want to take medicine using ANOVA.

We hypothesized that patients whose preferred treatment matched their current treatment would have less regret and higher SDM Process scores compared to patients whose treatment preference did not match their current treatment.

#### Brief depression-DQI version

A short version with 5 knowledge items was created based on item performance and discussion with experts in clinical content and survey methodology. We present data on retest reliability, reproducibility and examined known group validity for the brief version. The correlation between the 10- and 5-item versions was r = 0.76, *p* < 0.001.

All analyses were conducted in R version 3.6.0 [22] within RStudio version 1.1.4. [23].

## Results

### Patient and physician sample characteristics

In the patient sample, 405/504 (80%) completed the initial survey and 320/405 (79%) completed the retest. On average respondents were 40 years old (SD 13) and 61% were Caucasian. Patient characteristics are summarized in Table 1. Among the 200 physicians sent surveys, 13 were excluded (6 not eligible and 7 had an undeliverable address). Of those who were not excluded, 114/187 (60%) completed the survey and their characteristics are summarized in Table 2.

**Table 1:** Demographics of patients

	Total N = 405	DA (n = 191)	No DA (n = 214)	*p*-value
Gender (female)	266 (66%)	122 (64%)	144 (67%)	0.537
Age mean (SD)	40 (13)	39 (12.8)	41 (13.3)	0.218
Hispanic (Yes)	32 (8%)	17 (9%)	15 (8%)	0.76
Race				
White	238 (61%)	119 (63%)	119 (60%)	0.592
Black	103 (27%)	50 (26%)	53 (27%)	
Asian	13 (3%)	5 (3%)	8 (4%)	
Native Hawaiian/Pacific Islander	0 (0%)	0 (0%)	0 (0%)	
American Indian/Alaska Native	5 (1%)	1 (1%)	4 (2%)	
Other	4 (1%)	2 (1%)	2 (1%)	
Multiple	19 (5%)	9 (5%)	10 (5%)	
Not answered	23 (6%)	5 (3%)	18 (8%)	
Education				
College graduate or more	146 (36%)	72 (38%)	74 (35%)	0.84
Some college	162 (40%)	76 (40%)	86 (40%)	
High school or less	85 (21%)	43 (23%)	42 (20%)	
Not answered	12 (3%)	0 (0%)	12 (6%)	
Income				
< $30,000	218 (54%)	110 (58%)	108 (50%)	0.74
$30,001–60,000	89 (22%)	39 (20%)	50 (23%)	
$60,001–100,000	52 (13%)	26 (14%)	26 (12%)	
> $100,000	23 (6%)	12 (6%)	11 (5%)	
Not answered	23 (6%)	4 (2%)	19 (9%)	
Treatments in past 12 months				
Medicine	262 (70%)	123 (69%)	139 (71%)	0.787
Counseling or therapy	222 (59%)	106 (60%)	116 (59%)	0.979
Other	158 (40%)	78 (42%)	80 (38%)	0.527
Subjective numeracy scale	33 (9.5%)	33 (9%)	33 (10%)	0.763
Mental health composite score mean (SD)	34 (12)	33 (12)	35 (12)	0.056
Physical composite score mean (SD)	47 (13)	47 (12)	47 (13)	0.662

**Table 2:** Demographics of providers

	All providers	Primary care physicians	Psychiatrists
Gender, % male	76 (67%)	39 (74%)	38 (63%)
Age, mean (SD)	53 (8.2%)	52 (8.1%)	54 (8.2%)
Years in practice, mean (SD)	22 (9.1)	21 (9.7)	23 (8.8)
Patients seen per year with depression, median (IQR)	23 (15, 29)	22 (13, 30)	24 (15, 29)
Hispanic (yes)	2 (2%)	1 (2%)	1 (2%)
*Race*			
White	82 (75%)	38 (72%)	42 (75%)
Black	8 (7%)	4 (8%)	5 (9%)
Asian	16 (15%)	11 (21%)	5 (9%)
Other race	3 (3%)	0 (0%)	3 (5%)
Multiple races	1 (1%)	0 (0%)	1 (2%)
Missing	4 (4%)	0 (0%)	4 (7%)

### Item retention and deletion

*Knowledge*: Based on a priori criteria, a 10-item and 5-item knowledge scale were created. Patients’ total knowledge scores on the 10-item scale ranged from 14.6–100% with no evidence of a floor or ceiling effect (skew: 10-item =  − 0.24, 5-item =  − 0.04). The knowledge questions are presented in Additional file 1.

*Goals and concerns*: Of the seven goals, only one demonstrated evidence of a ceiling effect. For the item “to get relief from your symptoms of depression”, 62% selected 10 out of 10 (or extremely important) (Table 3). However, this goal was retained because a high percentage of patients selected this as one of their top two most important concerns. No items were eliminated because they were not the first or second choice. The remaining analyses were conducted using the full set of seven goals and concerns.

**Table 3:** Distribution of goals and concerns and ratings of importance

	M (SD)	Range	Ceiling (%)^a^	% top 1st or 2nd choice	% with problematic formats or missing	Retest ICC (95% CI)
To get relief from your symptoms of depression	8.9 (1.8)	0–10	62.5	71.6	0.5	0.67 (0.59, 0.74)
To feel better as quickly as possible	8.6 (1.9)	0–10	51.9	52.5	0.5	0.72 (0.65, 0.78)
To be able to return to your Regular activities	8.8 (1.8)	0–10	54.4	29.8	1.5	0.66 (0.57, 0.73)
To minimize out-of-pocket costs	7.9 (3)	0–10	51.5	13.4	1.7	0.65 (0.57, 0.72)
To avoid taking anti-depressant medicine	4.7 (3.5)	0–10	15.7	12.9	0.7	0.83 (0.78, 0.86)
To avoid the side effects of anti-depressant medicine	7.2 (3.1)	0–10	37.2	16.9	1	0.68 (0.6, 0.74)
To avoid going to depression counseling or therapy	4 (3.5)	0–10	11.5	2.7	1	0.76 (0.7, 0.81)

^a^Percent with a score of 10

#### Acceptability and feasibility

The overall response rate was high for the patient sample (80%). Overall, there were few missing items: 1.4% (range 0.2–7.4%). However, when we combined missing with problematic responses, all the knowledge items tripped the 5% threshold (range 5.9–11.6%). For example, of the 405 patients that viewed the item asking, “When do most side effects of anti-depressant medicine usually start?”, 257 chose the correct answer, 124 chose an incorrect response, 18 skipped the page, and 9 did not respond to the item. If we calculate the percent of unusable data (18 with skipped responses and 6 missing elements) this results in 5.9% (24/405); however, if we don’t take skipped responses into account, we find only 1.6% (6/405) missing data.

#### DQI-depression knowledge score

*Known group validity*: Table 4 describes the mean (SD) knowledge scores for patients and providers for the 10- and 5-item versions of the knowledge questions. Individual item difficulty for patients ranged from 15.3 to 96.3% for the 10-item and 15.3–91.7% for the 5-item. Across the versions, providers had higher mean knowledge scores than patients. Patients who received the DA had slightly higher mean knowledge scores than those who did not.

**Table 4:** Knowledge scores across samples for the two versions

Version	Patients (N = 386*) M (SD)	Providers (N = 114) M (SD)	*p*	DA (N = 187) M (SD)	No DA (N = 199*) M (SD)	*p*
10 items	63 (15)	81 (11)	< 0.001	64 (16)	61 (14)	0.041
5 items	53 (19)	68 (18)	< 0.001	55 (20)	52 (18)	0.043

*Total N = 385 in the 10-item version, No DA n = 198 in the 10-item version

*Retest reliability*: The 10-item version had retest reliability ICC = 0.65 (95% CI 0.53, 0.70); the 5-item had an ICC = 0.57 (95% CI 0.56, 0.72).

### DQI-goals and concerns

#### Known group validity

Univariate analyses for each of the seven goals and concerns predicting the choice for medication are presented (Additional file 2). Taking medication in the past 12 months was related to the goals of avoiding taking anti-depressant medicine (the higher this goal was rated the less likely the patient was to take medication), getting relief from your symptoms of depression (the higher this goal was rated the more likely the patient was to take medication), wanting to feel better as quickly as possible (the higher this goal was rated the more likely the patient was to take medication), and avoiding the side effects of anti-depressant medicine (the higher this goal was rated the less likely the patient was to take medication). No other goals were significant predictors (*p* > 0.07).

Based on results of the univariate analyses, four goals and concerns were selected for the multivariate logistic regression to predict taking medication (Table 5; alpha criterion set at 0.02). With all four predictors included, ‘feeling better as quickly as possible’ was not predictive (*p* = 0.261), ‘avoiding taking anti-depressants’ was predictive (those who rated this higher were less likely to take medications), and ‘avoiding side effects’ and ‘to get relief from your symptoms of depression’ were not significant (*p* = 0.35 and *p* = 0.74, respectively). The Hosmer Lemeshow goodness of fit test (*p* > 0.05 indicates a good fit) indicated the model does fit well, indicating that these measures of patients’ preferences do accurately predict whether or not they actually take medication (X^2^(8) = 11.24, *p* = 0.188).

**Table 5:** Multivariate model to generate predicted probability of taking antidepressant medicine based on patients’ goals

Factor	Taking medicine N = 256	Not taking medicine N = 59	Univariate *p*	Multivariate OR (95% CI)	*p*
To feel better as quickly as possible	8.7 (1.85)	8.3 (2.07)	0.062	1.1 (0.93, 1.29)	0.261
To avoid taking anti-depressant medicine	3.68 (3.25)	6.83 (3.22)	0	0.73 (0.66, 0.8)	0
To avoid the side effects of anti-depressant medicine	6.8 (3.29)	8.07 (2.75)	0	1.05 (0.95, 1.17)	0.35
To get relief from your symptoms of depression	9.05 (1.71)	8.59 (2.02)	0.026	1.03 (0.87, 1.22)	0.743
Intercept				3.08 (0.83, 12.15)	0.098

The ANOVA did distinguish among patients with different treatment preferences in the predicted direction F(2, 389 = 65.12, *p* = 0, η^2^ = 0.93, 0.25. The mean of the predicted probability indicates that those who wanted medication (M = 0.79, SD = 0.17) had the highest predicted probability of taking medication, followed by those who were unsure (M = 0.71, SD = 0.16), and those who did not want medication had the lowest predicted probability of taking medication (M = 0.56, SD = 0.19), all ps < 0.001.

#### Matching score

A little more than half of the sample received treatment that matched their preference (60.5%, Table 6). The majority of patients who wanted medication were on it (162/184, 88.0%; Table 6). However, the majority of patients (51/113, 52.0%) who did not want medication or who were unsure (45/73, 61.6%) also reported taking medication.

**Table 6:** Matching score

	Did not want medication	Want medication	Unsure	
Did not take medication	**62 (16.8%)**	22 (5.9%)	28 (7.6%)	112
Took Medication	51 (13.7%)	**162 (43.8%)**	45 (12.2%)	258
	113	184	73	370

Bolded groups received treatment that matched their preference

#### Retest reliability

The retest reliability for the treatment preference item was 0.66 (95% CI 0.57–0.73).

#### SDM process score

The average SDM Process score for the sample was 2.13 (SD = 1.00). There was no significant difference in SDM Process scores between those who matched on wanting/receiving medicine (M = 2.18, SD = 0.97), and those who did not (M = 2.06, SD = 1.07; t(320) =  − 1.06, *p* = 0.29).

#### Retest reliability

The SDM Process score had retest reliability of 0.71 (0.64–0.77).

#### Regret

Those who matched on wanting/receiving medicine had significantly lower regret scores compared to those who did not (M = 1.72, SD = 0.74 vs. M = 2.32, SD = 0.8; t(301) =  − 6.63, *p* < 0.001).

## Discussion

This research presents the development and initial evaluation of a new survey, the Depression Decision Quality Instrument (DQI), that measures the extent to which patients are informed about their condition and the treatment options, and if patients’ treatment reflects their preferences. The Depression DQI was acceptable to patients based on the low number of missing responses, was feasible for patients to self-administer and showed evidence of reliability and validity.

A high-quality health care decision has two components that require different types of assessments. The first is whether the patient is informed. Measuring patients’ knowledge typically requires questions that cover critical information about the disease and treatment options. A good knowledge test will be able to distinguish between those with high and low knowledge—and the 10 and 5-item versions were able to differentiate between patients and providers (providers had higher scores) and between patients who viewed a DA and those who did not (DA viewing had higher scores). Although statistically significant, the DA group did not have a meaningfully higher knowledge score than the non-DA group. For comparison, the Cochrane Systematic Review on use of DAs in people facing a health decision reported a 13% mean difference in those who received a DA versus those who did not [24], whereas we only found a mean difference of 2.8%. When compared to data from a systematic review on DAs specifically for adults with depression, the standardized mean difference in patient knowledge was 0.65 (0.14–1.15) compared to no decision aid [25], which differs from the standardized mean difference of 0.21 in this study.

The knowledge assessment highlighted some gaps in patients’ knowledge. For example, 83% of participants did not know that counseling and medicine are equally effective for patients with mild depression (the majority thought therapy was better), and 72% did not know that about 1/3 of people with moderate to severe depression who do not do anything to treat it will feel better within a year (majority thought it was higher). Possible reasons for the limited impact of the DA is that the DQI knowledge items were developed separately from the DA. The knowledge questions were not designed specifically to match the DA; however, the information needed to correctly answer the knowledge items was included in the DA. Also, patients who reported receiving the DA may have different knowledge than those who received and reviewed the DA. Whether or not patients received a DA, there is still a significant knowledge deficit for patients in the sample, which is similar to a national study that found patients had inadequate knowledge about depression treatment [13].

Understanding what matters to patients is necessary to determine if patients are receiving their preferred treatment. The goals and concerns were able to identify the variety of patient perspectives and they had acceptable test–retest reliability. We found the only goal that was predictive of taking medications was how important it was to avoid taking an anti-depressant medicine (those who wanted to avoid taking medication were less likely to be taking medication). About 50% in this sample wanted medication, which is in contrast to other studies [26, 27] where the majority of people preferred psychotherapy over medication. However, a recent survey of a nationally representative sample reported depression treatment preferences differed by race, ethnicity and gender with non-Hispanic white respondents preferring talk therapy over medication, but men preferring medication over talk therapy [28].

When we examined whether patients received their preferred treatment, a little over half did. Of those who wanted medication, 69% (120/173) were asked their preference for medication, 73% (68/93) of those who did not want medication were asked their preference, and 62% (37/60) who were unsure about medication were asked their preference. Notably, 14% of the sample were taking medication even though they did not indicate a preference for it. Prior work using goals and concerns to predict treatment choices utilized a predicted probability cutoff of 0.50 to distinguish between patients who wanted the treatment (≥ 0.50) and those who did not (< 0.50). However, given this data we find that all our groups—those who wanted medication, did not want medication, or were unsure—all had predicted probabilities greater than 0.50. Whether this finding represents the lack of attention that the medical system pays to patients’ preferences in selecting treatment for depression, a systematic bias toward the use of medication for treatment, or a limitation of the items used to measure preferences is not clear.

Many people with depression want to be informed about their condition and involved in treatment decisions [29], but the evidence supporting patient preference and treatment outcomes has been inconsistent [27]. Results from a systematic review and meta-analysis revealed that patients who received their preferred psychosocial mental health treatment resulted in lower treatment dropout rates and an improved therapeutic alliance [30]. For people considering depression treatment options, eliciting the patient’s treatment preference could provide valuable information to help the clinician provide care that may achieve improved outcomes. In fact, not eliciting and incorporating the patient’s preference can result in a type of medical error called a preference misdiagnosis [31].

Effective shared decision making requires asking patients what they prefer. The SDM Process scores were fairly low compared to other conditions [12, 20], but were similar to previously published SDM Process scores for depression treatment [13]. The patient responses on the individual items in the SDM Process scale revealed that 265 (80.8%) discussed options, 66 (20.1%) discussed pros (a lot or some), 28 (8.5%) discussed cons (a lot or some), and 228 (69.5%) were asked their preference; this is similar to how treatment decisions are discussed and framed [12]. There was no difference in the SDM Process score between those who did and didn’t want medicine, but we speculate that this may be because 70% of this sample was taking medicine.

Patients who received their preferred treatment had significantly less regret. The Cochrane systematic review on DAs supports this as, compared to usual care, patient DAs result in more people selecting options that are congruent with their informed values [24].

Previously published Decision Quality Instruments have focused on surgical decisions, which are one-time decisions. This project described the development of a tool to measure decision quality for depression treatment, which is a chronic condition. The challenge with measuring decision quality for chronic conditions is that patients and their physicians are likely to re-visit the discussion over time and treatments can be started and stopped, depending on circumstances. Overall, we felt that this tool was able to measure the two components of decision quality (knowledge and goals/concerns). The measure of knowledge differentiated between patients who received a DA, patients who did not receive a DA and physicians. Additionally, patients who wanted to avoid taking medication were more likely to not be on medication. This tool focused on depression medication treatment, but many patients will be offered both medication and counseling/therapy. Additional testing is needed to determine how well the Depression DQI operates if it is focused on counseling, or both medication and counseling. Future work should include measuring decision quality for other chronic conditions, as well as exploring the retrospective and prospective changes in the components of the DQI. Collecting data on moderating variables, such as availability of treatments, severity of symptoms or the impact on quality of life might be useful to better describe the conversation between a patient and health care provider because it would provide more detail than just taking medication or not. Lastly, the wording of items may need to be evaluated and adjusted for cultural meaning (for example, in some countries, medication is considered therapy), if used outside the United States.

The study has several limitations. Though the study was conducted in 2010, the prevalence of depression in US adults between 2007–2008 and 2015–2016 did not change significantly [32]. Recall bias is a possibility because patients were asked about a decision they made in the past year. Most of the patients in this sample were taking medication, so it will be important to explore these questions in a population more evenly split on depression treatment options. Data was not available to describe the availability of treatments. The population studied included participants from 17 cities and was 39% non-Caucasian which indicates diversity in the population surveyed, but the sample did not include patients whose primary language was not English. A next step is to gather data on the use of the knowledge questions and goals and concerns prospectively to determine if they are a useful tool in clinical practice to help with shared decision making. The model used to test if the goals and concerns were predictive of treatment preference was limited by the sample where 70% were taking medication. This imbalance made it easier to predict those who are taking medication as compared to those who are not.

## Conclusions

The Depression DQI tool demonstrated modest reliability and good validity and can be used to measure how informed patients are and what matters to them about their treatment choices. This tool can be useful in clinical practice or research to determine if patients are informed about their treatment options for depression and if they are receiving the treatment they prefer.

## Supplementary Information

**Additional file 1**. Knowledge items.

**Additional file 2**. Univariate analyses predicting probability of taking medication.

**Additional file 3**. Analyses for counseling/therapy.

## Data Availability

The datasets used and/or analysed during the current study are available from the corresponding author on reasonable request.
