# Supplementary material for: Psychometric evaluation of a decision quality instrument for medication decisions for treatment of depression symptoms
Source: BMC Med Inform Decis Mak. 2021 Aug 27;21:252. doi: 10.1186/s12911-021-01611-w (PMC8394109; doi:10.1186/s12911-021-01611-w)
Supplement: Supplementary file 1 — Additional file 1. Knowledge items. [file 12911_2021_1611_MOESM1_ESM.docx]

Additional file 1. Knowledge Items

| **Item** | **Question and Response Options** |
| --- | --- |
| 1* | Out of 100 people whose health care provider told them they have moderate to severe depression and who do not do anything to treat it, about how many will feel substantially better within a year?   - Fewer than 15 - Between 16 and 40 (correct) - Between 41 and 60 - More than 60 |
| 2* | For most people who have been told they have mild depression, which treatment works better?   - Anti-depressant medicine - Depression counseling or therapy - There is little or no difference (correct) |
| 3* | For most people who have been told they have severe depression, which treatment works better?   - Anti-depressant medicine only - Depression counseling or therapy only - Combination of medicine and counseling or therapy (correct) |
| 4* | About how many weeks does it usually take a person with depression to feel the benefits of anti-depressant medicine?   - Less than 2 weeks - 2 to 8 weeks (correct) - 9 to 15 weeks - More than 15 weeks |
| 5 | When do most side effects of anti-depressant medicine usually start?   - Less than 2 weeks (correct) - 2 to 8 weeks - 9 to 15 weeks - More than 15 weeks |
| 6-a | Are sexual problems a common side effect for some anti-depressant medicines?   - Yes (correct) - No |
| 6-b | Is dry mouth a common side effect for some anti-depressant medicines?   - Yes (correct) - No |
| 6-c | Is weight gain a common side effect for some anti-depressant medicines?   - Yes (correct) - No |
| 6-d | Is hair loss a common side effect for some anti-depressant medicines?   - Yes (correct) - No |
| 7 | If a person tries anti-depressant medicine and does not feel better after 2 months, what should he or she do next?   - Stop taking the medicine - Continue taking the medicine for at least a year - Work with the doctor to try something different (correct) |
| 8* | If a person starts feeling better after taking anti-depressant medicine, when should he or she talk with the doctor about stopping the medicine?   - As soon as a person feels better - 4-12 months after feeling better (correct) - A person should never stop taking the medicine |
| 9 | If a person tries depression counseling or therapy and does not feel better after 3 months, what should he or she do next?   - Stop counseling or therapy - Continue counseling or therapy for at least one year - Work with the doctor to try something different (correct) |
| 10-a | Can depression be caused by how the brain works?   - Yes (correct) - No |
| 10-b | Can depression be caused by stresses in a person’s life, such as job loss or divorce?   - Yes (correct) - No |
| 10-c | Can depression be caused by a bad day at work?   - Yes - No (correct) |
| 10-d | Can depression be caused by an argument with a friend?   - Yes - No (correct) |

*Included in 5-item version
