# Supplementary material for: Psychometric evaluation of a decision quality instrument for medication decisions for treatment of depression symptoms
Source: BMC Med Inform Decis Mak. 2021 Aug 27;21:252. doi: 10.1186/s12911-021-01611-w (PMC8394109; doi:10.1186/s12911-021-01611-w)
Supplement: Supplementary file 2 — Additional file 2. Univariate analyses predicting probability of taking medication. [file 12911_2021_1611_MOESM2_ESM.docx]

Additional file 2. Univariate analyses predicting probability of taking medication

| Goal | b | se | t | p | OR |
| --- | --- | --- | --- | --- | --- |
| to get relief from your symptoms of depression | 0.03 | 0.01 | 2.23 | 0.026 | 1.03 |
| to feel better as quickly as possible | 0.02 | 0.01 | 1.87 | 0.062 | 1.02 |
| to be able to return to your regular activities | 0.01 | 0.01 | 1.1 | 0.272 | 1.01 |
| to minimize out-of-pocket costs | -0.01 | 0.01 | -1.08 | 0.281 | 0.99 |
| to avoid taking anti-depressant medicine | -0.05 | 0.01 | -8.55 | 0 | 0.95 |
| to avoid the side effects of anti-depressant medicine | -0.03 | 0.01 | -3.56 | 0 | 0.97 |
| to avoid going to depression counseling or therapy | -0.01 | 0.01 | -0.8 | 0.423 | 0.99 |
