# Supplementary material for: Psychometric evaluation of a decision quality instrument for medication decisions for treatment of depression symptoms
Source: BMC Med Inform Decis Mak. 2021 Aug 27;21:252. doi: 10.1186/s12911-021-01611-w (PMC8394109; doi:10.1186/s12911-021-01611-w)
Supplement: Supplementary file 3 — Additional file 3. Analyses for counseling/therapy. [file 12911_2021_1611_MOESM3_ESM.docx]

**Additional file 3. Analyses for Counseling/Therapy**

Multivariable logistic model predicting whether a patient is going to counseling/therapy (in past 12 months) using only significant goals.

| **Factor** | **Receiving Counseling/ Therapy**  **N=219** | **Not Receiving Counseling/ Therapy**  **N=96** | **Univariate p** | **Multivariate OR (95%CI)** | **p** |
| --- | --- | --- | --- | --- | --- |
| to avoid the side effects of anti-depressant medicine | 6.93 ( 3.3 ) | 7.57 ( 2.99 ) | 0.055 | 0.97 ( 0.9 , 1.04 ) | 0.439 |
| to avoid going to depression counseling or therapy | 3.06 ( 3.23 ) | 5.42 ( 3.46 ) | <.001 | 0.82 ( 0.77 , 0.87 ) | <.001 |
| Intercept |  |  |  | 4.15 ( 2.31 , 7.73 ) | <.001 |

Receiving counseling in the past 12 months was related to avoiding going to depression counseling or therapy (the higher this goal was rated the less likely the patient was to receive counseling). No other goals were significant predictors (all ps>.2).

The ANOVA distinguished among patients with different treatment preferences. Using current use of counseling/therapy to predict the predicted probabilities of receiving counseling/therapy does reach significance F(2,391)=32.98,p<.001, eta2=0.92, 0.14. Those who wanted therapy (M=0.64, SD=0.15), were more likely to receive therapy than those who were unsure (M=0.58, SD=0.15), and both were more likely to receive therapy than those who did not want therapy (M=0.5, SD=0.16), all p<.001.

Matching Score:

Matching Score for Counseling/Therapy in the past 12 months

|  | **Don't want therapy** | **Want therapy** | **Unsure** |  |
| --- | --- | --- | --- | --- |
| Don't get therapy | **68 (18.3%)** | 50 (13.5%) | 34 (9.2%) | 152 |
| Get therapy | 35 (9.4%) | **160 (43.1%)** | 24 (6.5%) | 219 |
|  | 103 | **210** | 58 | 371 |

*Bolded groups received treatment that matched their preference.

Test-retest reliability was assessed with the intra class correlation coefficient (ICC (2k)) with 95% confidence intervals (CIs) for counseling/therapy. The ICC was 0.50 (0.39, 0.58).

*Construct validity hypotheses:*

There was no difference in SDMP scores between those who matched on wanting/receiving counseling (M=2.25, SD=0.95), and those who did not (M=2.03, SD=0.88; t(243)=-1.8, p=0.073. When looking at the relationship between regret and SDMP for counseling, we do not find a significant correlation (r=-0.12, p=0.058), but the same relationship is present--as SDMP increased, regret decreases.
